# Economic Evaluation of Multilayer Silicone-Adhesive Polyurethane Foam Dressing for the Prevention of Pressure Ulcers in At-Risk Hospitalized Patients: US and Italian Perspective

**DOI:** 10.34172/ijhpm.8371

**Published:** 2024-12-16

**Authors:** Elisabetta Mezzalira, Elisa Ambrosi, Neil Askew, Leo Nherera, Richard Searle, Francis Fatoye, Cristiana Forni

**Affiliations:** ^1^Department of Diagnostics and Public Health, University of Verona, Verona, Italy.; ^2^Smith and Nephew, Fort Worth, TX, USA.; ^3^Department of Health Professions, Faculty of Health and Education, Manchester Metropolitan University, Manchester, UK.; ^4^IRCCS Istituto Ortopedico Rizzoli, Bologna, Italy.

**Keywords:** Cost-Effectiveness, Economic Evaluation, Pressure Ulcer, Pressure Injury, Prevention

## Abstract

**Background:**

Hospital-acquired pressure ulcers (HAPUs) constitute an important source of concern for healthcare systems due to their negative consequences on patient quality of life and hospital costs. This phenomenon is increasing worldwide, driven by an aging population and increasing prevalence of chronic conditions. This economic evaluation aimed to determine whether using a multilayer, silicone-adhesive polyurethane foam dressing shaped for the sacrum area, alongside standard prevention (SP), is cost-effective in preventing HAPUs for hospitalized patients compared to SP alone.

**Methods:**

We developed a decision-analytic model to estimate the expected costs and clinical benefits of using the polyurethane foam dressing from Italian and US payor perspectives. Model inputs were taken from published studies, and uncertainty was assessed using one-way sensitivity analysis (OWSA) and probabilistic sensitivity analysis (PSA).

**Results:**

From both US and Italian perspectives, using a foam dressing in addition to SP was found to be cost-saving in all hospital settings. That is, it reduced the incidence of HAPUs at a lower cost overall. The estimated savings were €179 per patient and $305 per patient from Italian and US perspectives. Following sensitivity analysis, the results remained cost-saving, suggesting that our findings are robust.

**Conclusion:**

This is the first economic analysis investigating the cost-effectiveness of preventive dressings and SP for avoiding sacral pressure ulcers for at-risk hospitalized patients. This analysis suggests that using a multilayer polyurethane foam dressing to prevent sacral HAPUs in at-risk hospitalized patients is a cost-effective strategy compared with SP alone and, therefore, should be considered as a strategy for PU prevention in hospital settings.

## Background

**Key Messages**

**Implications for policy makers**

- Implementing the multilayer, silicone-adhesive polyurethane foam dressing shaped for the sacrum area alongside standard prevention (SP) to avoid hospital-acquired pressure ulcers (HAPUs) is projected to result in substantial cost savings for hospitals in different patient populations. Policy-makers should consider adopting this strategy to use available resources efficiently.
- This study is backed by solid evidence highlighting that using the specified foam dressing improves patient outcomes and is cost-effective. Policy-makers can leverage this evidence to invest in interventions that reduce the economic burden and contribute to better health outcomes, reducing the incidence of the most common complication affecting hospitalized patients.
- It is important to recognize the long-term economic impact of preventing HAPUs, especially considering the aging population and the increase in chronic diseases. Adopting cost-effective prevention practices can contribute to the sustainability of healthcare systems from an economic, social, and environmental perspective.

**Implications for the public**

The research highlights that applying a foam dressing on specific parts of the body, specifically the sacrum area, for hospitalized and at-risk patients effectively prevents pressure ulcers in medical, surgical, and intensive care patients. This prevention approach improves patient outcomes and leads to significant cost savings for hospitals. This means improved care for the patients at risk of developing pressure ulcers, potentially avoiding complications and reducing the pressure on healthcare providers, saving resources that can be used for other purposes, and contributing to the healthcare system’s sustainability.

Pressure ulcers (PUs) have been defined as localized areas of tissue damage arising from excess pressure or pressure shear forces combined.^1^ PUs affect at least 2.5 million people in the United States alone, with incidence rates varying from 1.9% to 71.6% across different hospital settings and countries, such as Europe, the United States, and Canada.^2,3^ Zhang et al assessed the age-standardized prevalence and incidence rates for decubitus ulcers at a global level at 11.3 (95% confidence interval [CI]: 10.2-12.5) and 41.8 (95% CI: 37.8-46.2) per 100 000 population-years in 2019, respectively.^4^ These numbers highlight how PUs continue to constitute one of the significant threats to the sustainability of healthcare systems, not only because of the adverse clinical outcomes that correlate to patients’ health and quality of life but also due to their substantial impact in terms of use of resources and economic cost.^5^ In a 2019 study within the United States, Padula and Delarmente estimated the average national cost of hospital-acquired pressure ulcers (HAPUs), calculating the average cost of care as $10 708 per HAPU, with a total financial burden projected at $26.8 billion.^6^ In a similar study using real-world data, Wassel et al calculated the incremental cost of a single episode of a hospital-acquired pressure injury in the United States as $21 767 in 2014 prices.^7^ In Europe, particularly the United Kingdom, the mean cost of wound care in clinical practice across the National Health System in 2018 was estimated at £8720 per PU.^8^ However, this varied between £1382 per patient with a category 1 PU and >£8500 per patient with a category 2, 3, 4, or an unstageable PU. In Italy, Forni and Searle estimated the cost of treating a single PU episode in a population of older adults with hip fracture surgery to be around €6878.^9^ A systematic review from Demarré et al reported that the daily cost of pressure ulcer treatment per patient ranged from €1.71 to €470.49 across different settings. In contrast, the daily cost of prevention per patient ranged from €2.65 to €87.57 across all settings.^10^ The costs associated with HAPU treatment emphasize the need for effective prevention strategies. Forni et al and Chiari et al highlighted the importance of identifying predictive factors and implementing preventive measures such as bundle interventions for at-risk populations.^11,12^ In this context, over the last few years, there has been an increasing interest in using advanced dressings for HAPU prevention in addition to the gold standard of preventive measures.^13,14^ There is an expanding body of evidence demonstrating the effectiveness of soft silicone multi-layered foam dressings in preventing sacral and heel pressure ulcers in trauma and critically ill patients, emphasizing the role of innovative dressings in high-risk environments.^13,15,16^ Also, the “Multischiume” trial by Forni et al in 2022 provided evidence relating to the effectiveness of multilayer silicone-adhesive polyurethane foam dressings for sacral pressure ulcer prevention in at-risk hospital inpatients in medical, surgical, and intensive care units (ICUs) compared to standard of care alone, and highlighted the potential of these advanced dressings to reduce PUs in various clinical settings.^14^ However, it remains unclear whether using silicone layer polyurethane dressings for PU prevention is cost-effective in different hospital settings and whether the additional benefits from the dressings are sufficient to justify their use over and above PU prevention bundles alone on cost-effectiveness. Therefore, this economic evaluation aimed to determine whether the use of a multilayer, silicone-adhesive polyurethane foam dressing (ALLEVYN LIFE, Smith & Nephew, UK) alongside standard prevention (SP) for the prevention of HAPUs is a cost-effective strategy, compared with SP alone in hospitalized patients following a recently published randomized trial by Forni et al.^14^ The primary analysis separates Stage I and Stage ≥II HAPU incidence rates as reported by Forni et al. Using this clinical evidence, the study considers two separate costing perspectives applicable to an Italian or US payor’s perspective.

## Methods

###  Decision-Analytic Model Structure 

 We developed a decision tree model in Microsoft Excel to estimate the expected costs and benefits of a multilayer, silicone-adhesive polyurethane foam dressing used as an adjunct to SP, compared with SP alone to prevent pressure injuries in hospitalized patients. Data on pressure injury incidence was collected over a one-week period. However, the pressure ulcer treatment cost included the episode of care until a patient was discharged from an index hospitalization.^6,17^ The base case analysis used costing perspectives applicable to US and Italian healthcare settings and ran the models separately. These two settings were selected because they represented an example of different healthcare systems. Costs were uplifted to 2023 prices following the approach shown in Supplementary file 1, and since the time horizon is less than one year, no discounting was applied to the costs or benefits accrued. In Italy, there is a national healthcare system funded through public taxation. At the same time, in the United States, there is a private healthcare system where citizens pay directly with out-of-pocket payments or through private insurance.

**Figure 1:**
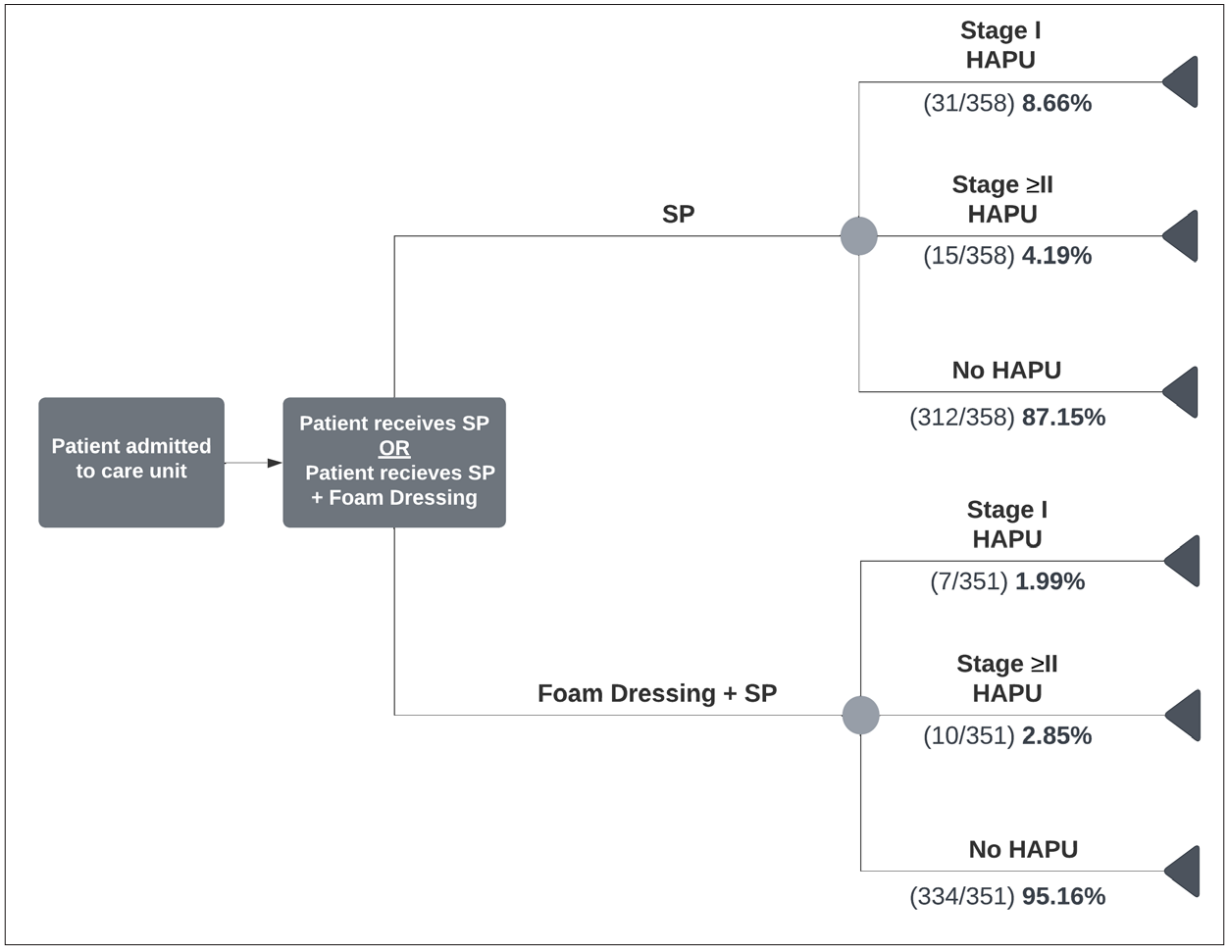
Decision-Analytic Model Structure of SP vs Foam Dressing + SP (Base Case HAPU Incidence). Abbreviations: SP, standard prevention; HAPU, hospital-acquired pressure ulcer.

Figure 1 displays the model structure, which shows the movement of patients in the model. This model structure is similar to a previously validated model by Forni and Searle in 2020 that assessed the cost-effectiveness of the same intervention for hip fracture patients.^9^ The main difference in the model structure used within this study is that HAPUs are separated into Stage I and Stage ≥II. When patients enter the model, they are treated with the foam dressing with SP or SP alone. Patients are exposed to the risk of developing a Stage I or Stage ≥II HAPU within 7 days of hospital admission. Otherwise, no pressure injuries would develop during the inpatient stay. The rate at which they develop HAPUs is governed by transitional probabilities, which were obtained from Forni et al.^14^ A rollback approach was used to calculate the expected costs and benefits for each tree branch, where the measure of benefit in this model was HAPUs avoided. Figure 1 shows the model structure for foam dressing in addition to SP, compared with SP alone, in preventing a HAPU in hospitalized patients.

###  Clinical Evidence for the Prevention of Hospital-Acquired Pressure Ulcers 

 This study used clinical data from a 2022 study by Forni et al, which conducted an open-label, parallel group, multi-center randomized controlled trial (RCT) that recruited 709 patients between October 2019 and March 2020 from 12 hospitals across 25 medical, surgical, and ICUs, involving specifically the following departments: orthopedics, urology, emergency surgery, general surgery, geriatrics, internal medicine, resuscitation, postoperative and cardiac intensive care, neurological and orthopedic rehabilitation, and cancer surgery.^14^ In detail, 27% of the patients came from the critical care area, 32% from the medical area, and 41% from the surgical area. Patients were, on average, 78 years old, had a mean Braden index of 13, and 44% were male. The different characteristics of patients and settings were well-balanced between the two study groups.

 The study reported that a multilayer foam dressing, when combined with SP, was effective in reducing the incidence of HAPU compared to SP alone. When separated by care setting, all groups remained statistically significant except when treatment was conducted in the ICU (5.2% SP + foam dressing vs 10.4% SP, *P* = .141).

 Our base case inputs for Italian and US perspectives, provided in Table 1, use clinical evidence across all care settings, which were shown to be statistically significant for Stage I (1.99% SP + foam dressing vs 8.66% SP, *P* ≤ .001). However, incidence rates for Stage ≥II HAPUs had a wider CI reported, and the difference was not statistically significant (2.85% SP + foam dressing vs 4.19% SP, *P* = .223).

**Table 1:** Base Case, One-Way Sensitivity Analysis, and Probabilistic Sensitivity Analysis Parameters

	**US (Mean & Base Case)**	**Italy (Mean & Base Case)**	**Range for OWSA**	**PSA Distribution**	**Used to Form PSA Parameters**	**PSA Distribution Inputs**	**Source**
**Dressing/Nurse Costs With Associated Resource Use**							
Foam dressing cost (12.9 × 12.9^2^)	$8.51	€2.94	+/- 20%	Normal	Assumed CV = 0.2	SD = $1.70 (US), €0.59 (Italy)	Average sales price
Nurse time cost per hour^a^	$40.85	€18.92	+/- 20%	Normal	Assumed CV = 0.2	SD = $8.17 (US), €3.78 (Italy)	Forni and Searle (2020)^9^
Dressing changes per foam dressing patient	1.7	1.7	+/- 20%	Gamma	Assumed SE = 20% of mean	Alpha = 25, Beta = 0.072	Forni et al (2022)^14^
No of minutes to change the dressing	15	15	+/- 20%	Normal	Assumed CV = 0.2	SD = 3 (US), 3 (Italy)	Forni and Searle (2020),^9^ Forni et al (2022)^14^
**Incidence of Pressure Ulcers (HAPUs)**							
Stage I HAPUs following SP	8.66%	8.66%	+/- 20%	Dirichlet	No. of events (n) and sample size (N)	Alpha (n) = 31, Sum of alpha (N) = 351	Forni et al (2022)^14^
Stage II HAPUs following SP	4.19%	4.19%	+/- 20%	Dirichlet	No. of events (n) and sample size (N)	Alpha (n) = 15, Sum of alpha (N) = 351	Forni et al (2022)^14^
Stage I HAPUs with SP & foam dressing	1.99%	1.99%	+/- 20%	Not varied in PSA	Not varied in PSA	Not varied in PSA	Forni et al (2022)^14^
Stage II HAPUs with SP & foam dressing	2.85%	2.85%	+/- 20%	Not varied in PSA	Not varied in PSA	Not varied in PSA	Forni et al (2022)^14^
**RR–PSA only**							
RR of developing Stage I HAPU (Foam dressing + SP vs SP alone)^b^	0.23	0.23	Not varied in OWSA	Lognormal	Reported CI (0.24, 0.79)	ln(mean) = -1.515 SE of ln(mean) = 0.304	Forni et al (2022)^14^
RR of developing Stage II HAPU (Foam dressing + SP vs SP alone)^b^	0.68	0.68	Not varied in OWSA	Lognormal	Reported CI (0.31, 1.49)	ln(mean) = -0.466 SE of ln(mean) = 0.401	Forni et al (2022)^14^
**Costs Resulting From a Pressure Ulcer (HAPU)**							
Cost to treat Stage I Pressure Ulcer (HAPU)^a^	$1719	€980	+/- 20%	Gamma	Assumed SE = 20% of mean	Alpha = 25 Beta = 68.78 (US), 39.21 (Italy)	Padula & Delarmente 2019 (US),^6^ calculated from Posnett et al 2009 (Italy)^17^
Cost to treat Stage II Pressure Ulcer (HAPU)^a^	$16 729	€9537	+/- 20%	Gamma	Assumed SE = 20% of mean	Alpha = 25 Beta = 669.17 (US), 381.49 (Italy)	Padula & Delarmente 2019 (US),^6^ calculated from Posnett et al 2009 (Italy)^17^

Abbreviations: SP, standard prevention; HAPU, hospital-acquired pressure ulcer; RR, relative risk; OWSA,one-way sensitivity analysis; PSA, probabilistic sensitivity analysis; CV, coefficient of variation; SE, standard error; SD, standard deviation (where SD equals the mean multiplied by the assumed coefficient of variation). ^a^ Please note that nurse time and pressure ulcer treatment costs were uplifted to 2023 using ISTAT (Italy) and BLS (US) data.^18,19^ ^b^ Relative Risk = 1 – Relative Risk Reduction.

###  Unit Costs 

 The cost of treating a HAPU in Italy was obtained from a published study that considered the cost-effectiveness of a foam dressing from Italian and US perspectives in older patients with hip fractures.^9,17^ From the Italian perspective, the cost of treating a pressure ulcer was adjusted to 2023 prices using healthcare inflation indices for Italy (Italian National Institute of Statistics, ISTAT) to give a unit cost of €980 and €9537 for Stage I and Stage ≥II HAPUs, respectively.^18^ This reflected an average hospitalization cost for treating a pressure ulcer in Italy.^17^

 The model used Padula and Delarmente’s simulation study, which estimated the cost of treating a Stage I and Stage II HAPU in the United States at $1452 and $14 126, respectively.^6^ When uplifted to 2023 prices using the Bureau of Labor Statistics (BLS) database, this resulted in model inputs of $1719 and $16 729 for treating Stage I and Stage II in the United States.^19^

 Intervention costs included the cost of the dressings used and the nursing time associated with the dressing application. The cost of dressing application was estimated by multiplying the time in minutes used to change the dressing by the unit cost per hour of staff time and the number of dressings used per patient. Estimates of the time taken to change a dressing and the number of dressing changes were obtained from a previous economic evaluation by Forni and Searle and the recent 2022 multi-center RCT by Forni et al.^9,14^

###  Scenario and Sensitivity Analyses 

 The base case model also uses point estimates obtained from published literature, which is subject to uncertainty. To investigate the effect of this uncertainty in the model inputs, we performed both One-way sensitivity analysis (OWSA) and probabilistic sensitivity analysis (PSA). OWSA includes changing model parameters one at a time and evaluating the model results using each parameter’s upper and lower limit estimates. This approach helps in assessing which parameters have the highest impact on the model results or model drivers. The results of the OWSA are presented as tornado diagrams. We also implemented a PSA, also known as a Monte Carlo simulation, which assigns a probability distribution to each model parameter and varies them simultaneously. Our model was run 1000 times to create 1000 pairs of incremental costs and pressure ulcers/HAPUs avoided, which is graphically presented as cost-effectiveness planes in the results section.

 The Base model used US cost data from Padula and Delarmente.^6^ In a scenario analysis, the cost of treating a HAPU in the United States was obtained from a retrospective study of a large US hospital database. This found the incremental cost of treating an average pressure ulcer as part of an index hospitalization to be $14 589 and $20 980 for Stage I and Stage II HAPUs, respectively. These estimates were adjusted for healthcare inflation using the BLS to produce model inputs of $18 402 and $26 465.^19^ From the Italian perspective, it was assumed that cost differences across HAPU stages would follow the same distribution. After adjusting Posnett and colleagues’^17^ estimated cost of treating an average HAPU in Italy of €6878, cost estimates for Stage I and Stage II HAPUs were €4610 and €6629, respectively adjusted to 2023 prices.^6^

## Results

###  Base Case Results 

 Prophylactic use of a multilayer, silicone-adhesive polyurethane foam dressing used as an adjunct to SP, compared with SP alone, in the prevention of pressure injuries in hospitalized patients was found to be cost-saving from both the Italian and the US payor perspectives. A cost-saving finding means that using the foam dressing in addition to SP resulted in less total cost and avoided more HAPUs compared to SP alone. Table 2 shows that for a cohort of 1000 patients, the foam dressing would result in 80 fewer HAPUs, saving €179 per patient for Italy and $305 per patient in the United States, respectively.

**Table 2:** Deterministic Results Per 1000 Patients (Italian and US Perspectives)

**Outcome**	**SP**	**SP + Foam Dressing**	**Difference/Saving**
Total number of foam dressing applications	0	1700	+1700
Number of nurse minutes	0	27 000	+27 000
Number of Stage I HAPUs	86	20	-66
Number of Stage II HAPUs	42	28	-14
Total number of HAPUs	128	48	-80
Total costs (€) – Italy	€484 486	€305 071	-€179 415
Total costs ($) – US	$849 838	$544 604	-$305 234
Base Case Decision Rule – Italy & US	SP with foam dressing dominates SP (fewer HAPUs at a lower cost)		

Abbreviations: SP, standard prevention; HAPU, hospital-acquired pressure ulcer.

###  One-Way Sensitivity Analysis 

**Figure 2:**
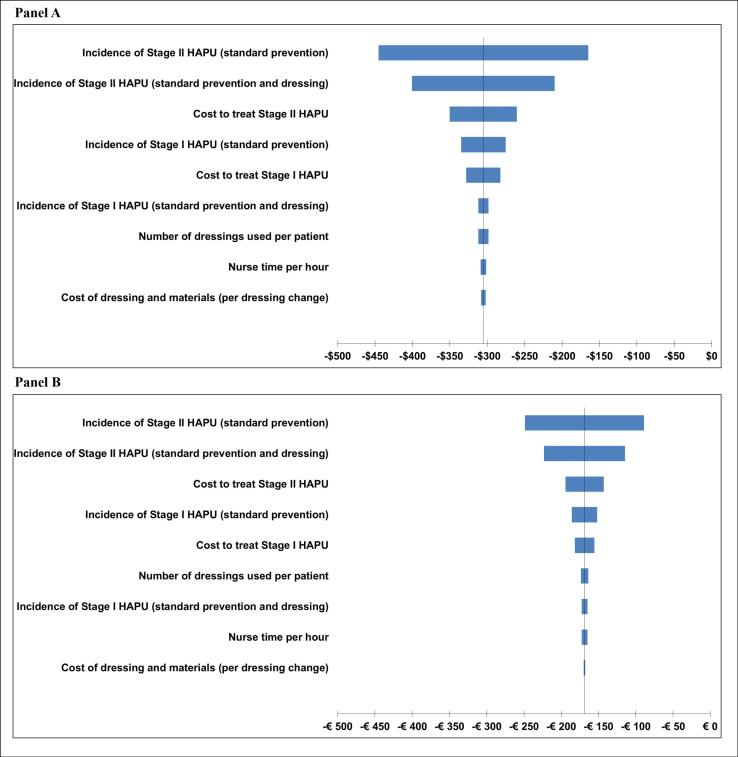
One-Way Sensitivity Analysis Results From US (Panel A) and Italian (Panel B) Perspective. Abbreviation: HAPU, hospital-acquired pressure ulcer.

 Results of OWSA are presented as tornado diagrams in in panels A and B of Figure 2 from a US and Italian perspective, respectively. The larger the bars on the tornado diagram, the more significant the impact of the parameter on the overall results. The plot shows that the model is sensitive to the incidence of HAPU (both SP and foam dressing + SP) and the cost of treating a HAPU. However, the model conclusions did not change when various model inputs were changed, with the foam dressing and SP remaining more effective and cost-saving. This finding suggests that our model results are robust and not due to chance.^19^

 Using the scenario of different costs for pressure injuries, we observed higher savings from the US and Italian’s perspective. The estimated savings are $1548 for the US perspective and €403 for the Italian perspective. The deterministic results for a cohort of 1000 patients can be found in Supplementary file 2. The probabilistic analysis and cost-effectiveness planes for this scenario can be found in Supplementary file 3.

###  Probabilistic Sensitivity Analysis 

**Figure 3:**
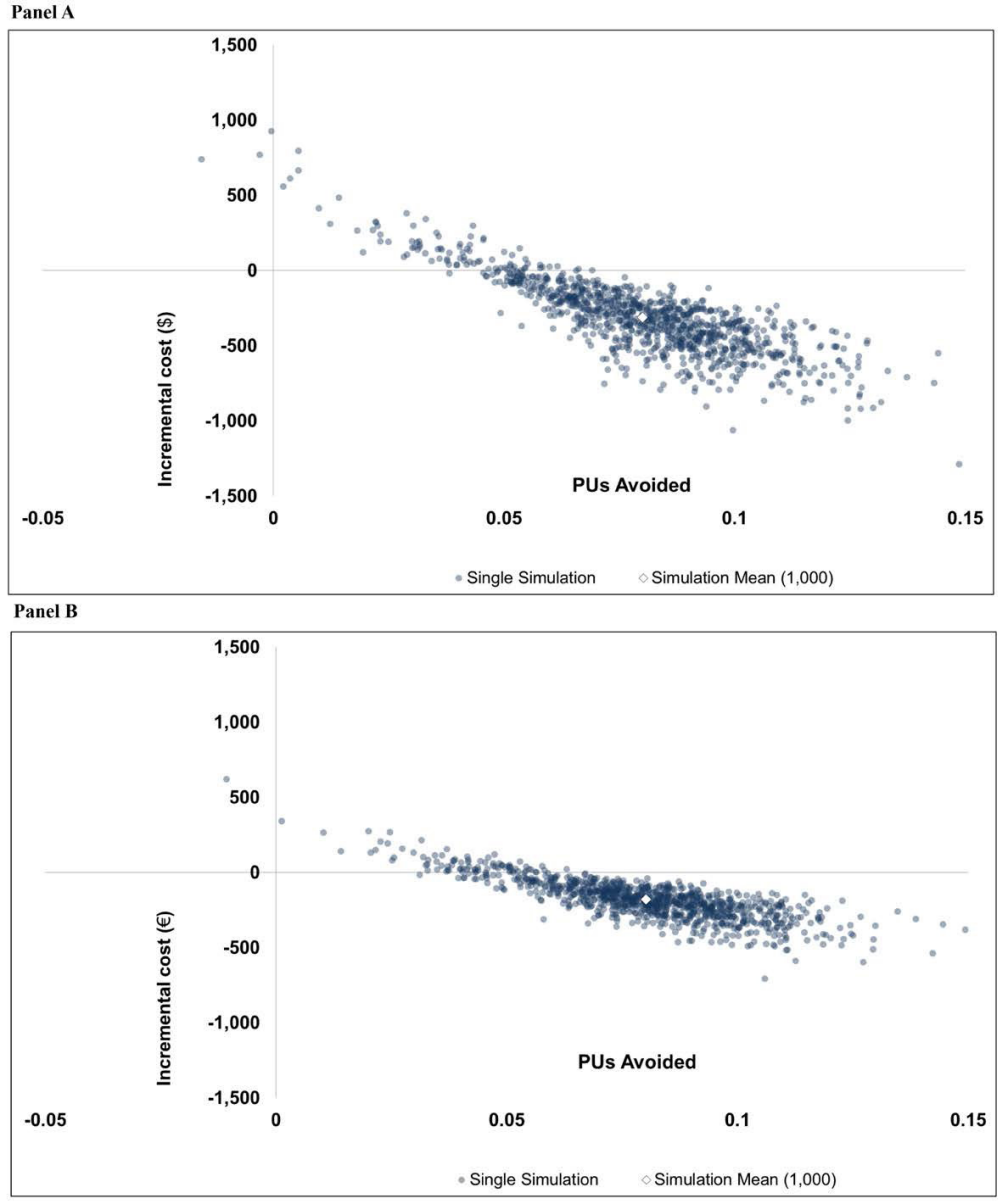
Cost-Effectiveness Plane With Probabilistic Sensitivity Analysis Results From US (Panel A) and Italian (Panel B) Perspective. Abbreviation: PUs, pressure ulcers.

 The probabilistic analysis results are presented graphically as cost-effectiveness planes in panels A and B of Figure 3 for the US and Italian perspectives, respectively. Figure 3 shows that using a foam dressing dominates SP by reducing total cost and the number of HAPUs in most of the 1000 simulations (91% = US, 92% = Italy). These simulations fall in the south-east quadrant of the cost-effectiveness plane and do not require a willingness-to-pay (WTP) threshold to demonstrate that adding a foam dressing is beneficial as they reduce both total cost and HAPUs. For simulations that fall in the north-east region (higher total cost and fewer HAPUs), a WTP threshold can be used to consider the proportion of cost-effective simulations at the selected WTP threshold. While the WTP threshold will differ by payor/provider, a conservative WTP threshold of $5000/€4500 demonstrated that 96.80% (US) and 98.70% (Italy) of simulated results show that the additional foam dressing is either a dominant or cost-effective treatment strategy. In addition, 95% CIs and minimum/maximum values from the simulated results are provided in Table 3.

**Table 3:** Probabilistic Sensitivity Analysis Results Per 1000 Patients From Across All At-Risk Hospitalized Patients (Italian and US Perspectives)

**Outcome**	**Min. **	**Lower 95% CI**	**Probabilistic Mean**	**Upper 95% CI**	**Max.**
Total HAPUs avoided – Italy	-11	36	80	118	149
Difference in Cost – Italy	€622 108	€81 673	-€179 827	-€444 780	-€705 456
Total HAPUs avoided – US	-16	31	80	122	149
Difference in Cost – US	$928 068	$197 793	-$310 567	-$753 418	-$1 284 084

Abbreviations: HAPU, hospital-acquired pressure ulcer; CI, confidence interval.

## Discussion

 Healthcare systems are challenged to provide quality care while maintaining a sustainable performance.^20^ Achieving sustainability in a healthcare system necessitates effective performance across a triple bottom line of financial, social, and environmental dimensions, which is a challenging goal to meet over time consistently.^21^ The application of successful prevention interventions is critical, so it is becoming increasingly important to conduct cost-effectiveness analyses to prevent adverse events. To our knowledge, no economic analysis has investigated the cost-effectiveness of preventive dressings and SP for avoiding sacral pressure ulcers for at-risk patients across medical, surgical, and intensive care hospital settings. This is the first analysis to demonstrate that the use of prophylactic foam dressings in addition to SP is cost-saving when compared to SP alone in preventing HAPUs when considering the perspective of two different healthcare systems presenting different organizations and funding sources, namely Italy and the US. Our findings confirmed what has been previously found by Forni et al, who conducted an economic evaluation on the cost-effectiveness of prophylactic foam dressings in addition to SP in at-risk patients over the age of 65 with hip fractures.^9^ The results of the present study extend the effectiveness and cost-effectiveness of prophylactic dressings for PU prevention to all at-risk inpatients across surgical and medical care settings. However, the intervention did not reach statistical significance for intensive care settings. However, Santamaria et al conducted an RCT to examine the cost-effectiveness of prophylactic dressings for PU prevention in critically ill patients in Australia. They found that the intervention was cost-saving since the average net cost of the intervention was lower than that of the control (AU$ 70.82 vs AU$ 144.56).^13^ Another cost-effectiveness analysis from a European RCT of critically ill patients found that preventive dressings were cost-effective for the sacral area in critically ill patients while marginally cost-effective for the heel area.^22^

 A systematic review from Demarré et al reported that the cost of pressure ulcer treatment per patient per day ranged from €1.71 to €470.49 across different settings. In contrast, the cost of prevention per patient per day ranged from €2.65 to €87.57 across all settings.^10^ As discussed by Demarré et al, and as highlighted by other studies, compared to the cost of treating PUs and their known association with negative patient outcomes, the cost of application of the adhesive preventive dressings can be considered relatively low.^9,10,13^ However, it is important to consider the adhesiveness of the chosen dressing and, consequently, the number of dressings needed for each patient during the hospitalization. As previously experienced and reported by Forni and Searle,^9^ the adhesive layer of the dressing used in this multi-center RCT reduces the burden on resources, reducing costs and waste materials and their associated carbon emissions. Interestingly, Makoto et al reported that multilayer silicone foam dressings can prevent sacrum and coccyx pressure ulcers in patients with persistent severe diarrhea and/or fragile skin, demonstrating their effectiveness in complex clinical and hygienic conditions.^23^ The capacity of foams to maintain their preventive proprieties in extreme environments (such as skin humidity) confirms their potential for HAPU prevention in at-risk hospitalized populations. Beeckman et al in 2021 found a reduced incidence of HAPUs of category 2 or higher in hospitalized at-risk patients using silicone foam dressings in addition to standard of care.^16^ However, while the intervention was effective for the sacrum area, the study found no statistical difference for the heel and trochanter areas. In another study, a 2023 systematic review and meta-analysis found that silicone dressings consistently reduce the incidence of PUs in intensive and non-intensive care settings, regardless of the type of dressing used.^24^ Given the costs of HAPU treatments for at-risk patients highlighted by our study, further studies should consider the cost-effectiveness of adding a foam dressing to heel and trochanter areas to prevent HAPUs to see if our findings hold for other areas of the body. The global prevalence of HAPUs and their consequences highlight the need for further research on this topic to improve patients’ outcomes and quality of life and support hospital managers and policy-makers with evidence for effective resource management.

 A significant strength of the model is that this is the first study to conduct a cost-effectiveness analysis using level one evidence from an RCT conducted in multiple hospital centers, with patients admitted in medical, surgical, and ICU settings. Moreover, our study used a previously validated model structure and has findings similar to other published studies, concluding that preventative dressings are cost-effective or cost-saving. We tested our model assumption in one-way and PSA and, in all cases, found that our model results were robust and not due to chance. In terms of limitations, it is important to highlight that the cost-effectiveness analysis is based on the results of a single study. However, the study is robust and of high quality. Our model focused on the difference in HAPUs between the two interventions in hospitalized patients. Therefore, while we present a simplified patient pathway, this was appropriate given that the focus was on modeling the impact of reducing pressure ulcers. Furthermore, the study was performed from the perspectives of Italian and US payors. Thus, while our results may apply to countries that deliver care through different healthcare systems, we caution that our results may not be comprehensively generalizable to every region. Therefore, to apply to other regions, cost-effectiveness should be considered using local cost data.

## Conclusions

 This analysis suggests that using a multilayer polyurethane foam dressing with SP to prevent sacral HAPUs in hospitalized patients is a cost-saving strategy compared with SP alone. The model conclusions did not change in sensitivity analyses, suggesting robust findings. Policy-makers and payers should consider the prophylactic use of foam dressing in addition to SP to prevent pressure ulcers in hospitalized patients.

## Ethical issues

 Secondary analysis of data obtained through the Multischiume RCT. Ethical approval was granted by the health service and university human research ethics committees of each institution involved in the study for the purpose of the RCT: Principal investigator (P.I.) center approval CE AVEC 41/2019/DISP/IOR.

## Conflict of interests

 Smith & Nephew has agreed to supply the dressings required for the RCT at no cost to all study participants. This arrangement has been made without influencing any stage of the research project. Additionally, Smith & Nephew has supported the conduction of the subsequent cost analysis under the same terms, maintaining the integrity and independence of the study outcomes. Smith & Nephew will cover the costs associated with open-access publication for this study.

## Supplementary files

Supplementary file 1. Example Calculation to Uplift Costs to 2023 Prices.

Supplementary file 2. Deterministic Results Using Data for HAPU Treatment Cost from a Large US Database.

Supplementary file 3. Probabilistic Results Using Data for HAPU Treatment Cost from a Large US Database.
