# Supplementary material for: Economic Evaluation of Multilayer Silicone-Adhesive Polyurethane Foam Dressing for the Prevention of Pressure Ulcers in At-Risk Hospitalized Patients: US and Italian Perspective
Source: Int J Health Policy Manag. 2024 Dec 16;13:8371. doi: 10.34172/ijhpm.8371 (PMC11806223; doi:10.34172/ijhpm.8371)
Supplement: Supplementary file 1 — Example Calculation to Uplift Costs to 2023 Prices. [file ijhpm-13-8371-s001.pdf]

Article title: Economic Evaluation of Multilayer Silicone-Adhesive Polyurethane Foam Dressing for the Prevention of Pressure Ulcers in At-Risk Hospitalized Patients: US and Italian Perspective
Journal name: International Journal of Health Policy and Management (IJHPM)
Authors' information: Elisabetta Mezzalira[1]*, Elisa Ambrosi[1], Neil Askew[2], Leo Nherera[2], Richard Searle[2], Francis Fatoye[3], Cristiana Forni[4]
[1]Department of Diagnostics and Public Health, University of Verona, Verona, Italy.
[2]Smith and Nephew, Fort Worth, TX, USA.
[3]Department of Health Professions, Faculty of Health and Education, Manchester Metropolitan University, Manchester, UK.
[4]IRCCS Istituto Ortopedico Rizzoli, Bologna, Italy.
***Correspondence to:** Elisabetta Mezzalira; Email: elisabetta.mezzalira@univr.it
Citation: Mezzalira E, Ambrosi E, Askew N, Nherera L, Searle R, Fatoye F, Forni C. Economic evaluation of multilayer silicone-adhesive polyurethane foam dressing for the prevention of pressure ulcers in at-risk hospitalized patients: US and Italian perspective. Int J Health Policy Manag. 2024;13:8371. doi:10.34172/ijhpm.8371
Supplementary file 1. Example Calculation to Uplift Costs to 2023 Prices

TABLE A1: Deterministic results per 1,000 patients (Italian and US perspectives)

**Outcome**	**SP**	**SP + Foam Dressing**	**Difference/saving**
Total number of foam dressing applications	0	1700	+1700
Number of nurse minutes	0	27000	+27000
Number of Stage I HAPUs	86	20	-66
Number of Stage II HAPUs	42	28	-14
Total number of HAPUs	128	48	-80
Total costs (€) – Italy	€711,490	€308,942	**-€402,548**
Total costs ($) – US	$2,702,385	$1,154,679	**-$1,547,706**
Base Case Decision Rule – Italy & US	SP with foam dressing dominates SP (fewer HAPUs at a lower cost)		
