# Supplementary material for: Economic Evaluation of Multilayer Silicone-Adhesive Polyurethane Foam Dressing for the Prevention of Pressure Ulcers in At-Risk Hospitalized Patients: US and Italian Perspective
Source: Int J Health Policy Manag. 2024 Dec 16;13:8371. doi: 10.34172/ijhpm.8371 (PMC11806223; doi:10.34172/ijhpm.8371)

Article title: Economic Evaluation of Multilayer Silicone-Adhesive Polyurethane Foam Dressing for the Prevention of Pressure Ulcers in At-Risk Hospitalized Patients: US and Italian Perspective

Journal name: International Journal of Health Policy and Management (IJHPM)

Authors' information: Elisabetta Mezzalira[1]*, Elisa Ambrosi[1], Neil Askew[2], Leo Nherera[2], Richard Searle[2], Francis Fatoye[3], Cristiana Forni[4]

[1]Department of Diagnostics and Public Health, University of Verona, Verona, Italy.

[2]Smith and Nephew, Fort Worth, TX, USA.

[3]Department of Health Professions, Faculty of Health and Education, Manchester Metropolitan University, Manchester, UK.

[4]IRCCS Istituto Ortopedico Rizzoli, Bologna, Italy.

***Correspondence to:** Elisabetta Mezzalira; Email: elisabetta.mezzalira@univr.it

Citation: Mezzalira E, Ambrosi E, Askew N, Nherera L, Searle R, Fatoye F, Forni C. Economic evaluation of multilayer silicone-adhesive polyurethane foam dressing for the prevention of pressure ulcers in at-risk hospitalized patients: US and Italian perspective. Int J Health Policy Manag. 2024;13:8371. doi:10.34172/ijhpm.8371

Supplementary file 2. Deterministic Results Using Data for HAPU Treatment Cost from a Large US Database

Panel A

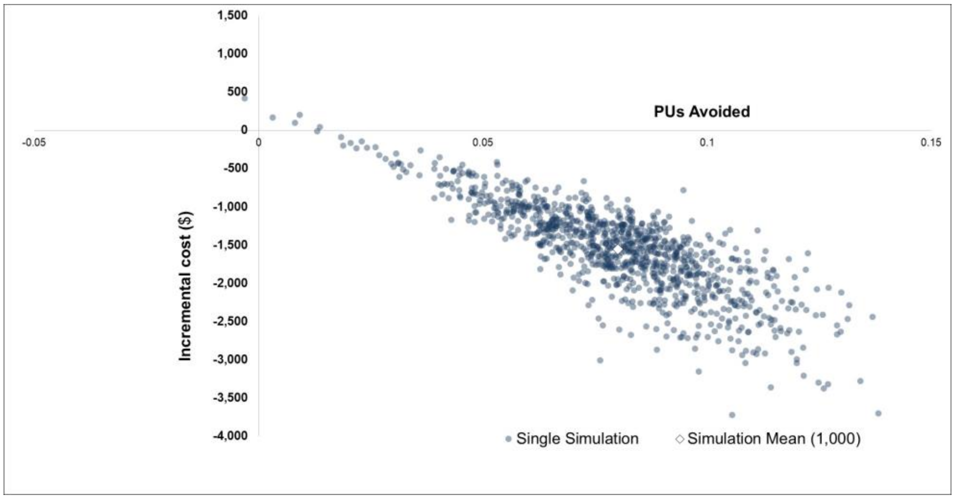

Panel B

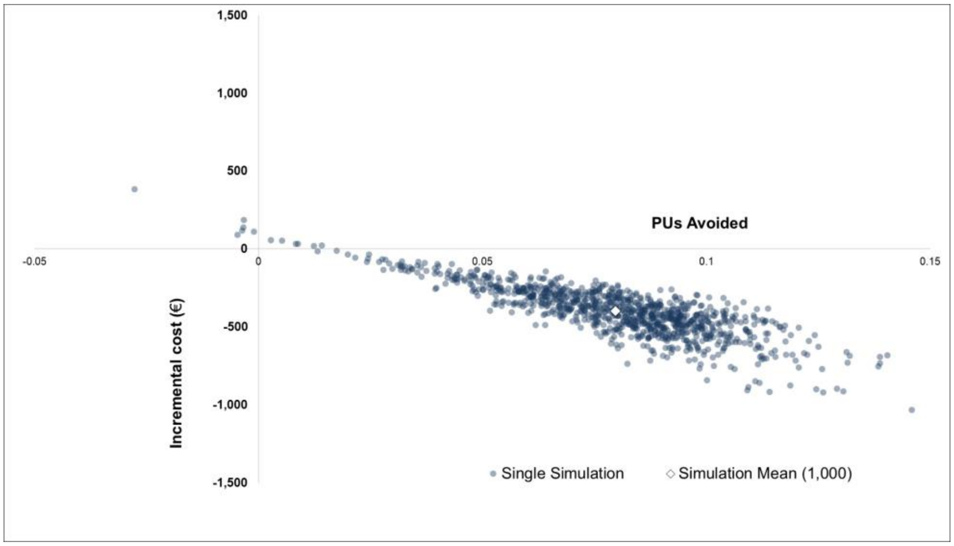

Supplement: Supplementary file 2 — Deterministic Results Using Data for HAPU Treatment Cost from a Large US Database. [file ijhpm-13-8371-s002.pdf]
