# Supplementary material for: Economic Evaluation of Multilayer Silicone-Adhesive Polyurethane Foam Dressing for the Prevention of Pressure Ulcers in At-Risk Hospitalized Patients: US and Italian Perspective
Source: Int J Health Policy Manag. 2024 Dec 16;13:8371. doi: 10.34172/ijhpm.8371 (PMC11806223; doi:10.34172/ijhpm.8371)
Supplement: Supplementary file 3 — Probabilistic Results Using Data for HAPU Treatment Cost from a Large US Database. [file ijhpm-13-8371-s003.pdf]

Article title: Economic Evaluation of Multilayer Silicone-Adhesive Polyurethane Foam Dressing for the Prevention of Pressure Ulcers in At-Risk Hospitalized Patients: US and Italian Perspective

Journal name: International Journal of Health Policy and Management (IJHPM)

Authors' information: Elisabetta Mezzalira[1]*, Elisa Ambrosi[1], Neil Askew[2], Leo Nherera[2], Richard Searle[2], Francis Fatoye[3], Cristiana Forni[4]

[1]Department of Diagnostics and Public Health, University of Verona, Verona, Italy.

[2]Smith and Nephew, Fort Worth, TX, USA.

[3]Department of Health Professions, Faculty of Health and Education, Manchester Metropolitan University, Manchester, UK.

[4]IRCCS Istituto Ortopedico Rizzoli, Bologna, Italy.

***Correspondence to:** Elisabetta Mezzalira; Email: elisabetta.mezzalira@univr.it

Citation: Mezzalira E, Ambrosi E, Askew N, Nherera L, Searle R, Fatoye F, Forni C. Economic evaluation of multilayer silicone-adhesive polyurethane foam dressing for the prevention of pressure ulcers in at-risk hospitalized patients: US and Italian perspective. Int J Health Policy Manag. 2024;13:8371. doi:10.34172/ijhpm.8371

Supplementary file 3. Probabilistic Results Using Data for HAPU Treatment Cost from a Large US Database

In this appendix, the authors show how prices were uplifted, using the incremental hospital cost of treating a pressure ulcer in the U.S. as an example.

The additional cost of treating a hospital-acquired pressure ulcer (HAPU) in the US was obtained from Wassel et al. (reference 7). This study estimated that for Stage I HAPUs, the index hospitalization cost would increase by $14,589 when a pressure ulcer occurs. This study presented costs in 2014 prices.

To update prices to 2023, we refer to the U.S. Bureau of Labor Statistics (BLS) Consumer Price Inflation (CPI) dataset (reference 17). Here, we obtained the following inflation series: "Medical care in U.S. city average, all urban consumers." The series ID is CUUR0000SAM.

It provides an inflation index with the base year set to 1982-1984=100. The authors re-based this index on the source year of the input, which, in this case, is 2014. Referring to Table C1, the 2023

value is calculated using the raw index as follows: (2014index/2023index) * 100. This results in (435.3/549.1) * 100 = 126.1.

Therefore, medical care costs have risen by 26.1% since 2014. We now multiply the raw cost value of $14,767 by 1.26142 to obtain $18,403, which serves as our Stage I HAPU treatment cost for the US perspective in 2023 prices.

Table C1 – Inflation upfit using series CUUR0000SAM from BLS database.

	Inflation base year	
**Year**	**1982-1984=100**	**2014=100**
**1982**	92.5	21.3
**1983**	100.6	23.1
**1984**	106.8	24.5
**1985**	113.5	26.1
**1986**	122.0	28.0
**1987**	130.1	29.9
**1988**	138.6	31.8
**1989**	149.3	34.3
**1990**	162.8	37.4
**1991**	177.0	40.7
**1992**	190.1	43.7
**1993**	201.4	46.3
**1994**	211.0	48.5
**1995**	220.5	50.7
**1996**	228.2	52.4
**1997**	234.6	53.9
**1998**	242.1	55.6

**1999**	250.6	57.6
**2000**	260.8	59.9
**2001**	272.8	62.7
**2002**	285.6	65.6
**2003**	297.1	68.3
**2004**	310.1	71.2
**2005**	323.2	74.2
**2006**	336.2	77.2
**2007**	351.1	80.6
**2008**	364.1	83.6
**2009**	375.6	86.3
**2010**	388.4	89.2
**2011**	400.3	92.0
**2012**	414.9	95.3
**2013**	425.1	97.7
**2014**	435.3	**100.0**
**2015**	446.8	102.6
**2016**	463.7	106.5
**2017**	475.3	109.2
**2018**	484.7	111.4
**2019**	498.4	114.5
**2020**	518.9	119.2
**2021**	525.3	120.7
**2022**	546.6	125.6
**2023**	549.1	**126.1**
